# Kinetics, Thermodynamics and Equilibrium Studies for Gold Recovery from Diluted Waste Solution

**DOI:** 10.3390/ma14185325

**Published:** 2021-09-15

**Authors:** Adina Negrea, Sylwia Ronka, Mihaela Ciopec, Narcis Duteanu, Petru Negrea, Maria Mihailescu

**Affiliations:** 1 Faculty of Industrial Chemistry and Environmental Engineering, Politehnica University of Timisoara, 2 Piata Victoriei, 300006 Timisoara, Romania; adina.negrea@upt.ro (A.N.); petru.negrea@upt.ro (P.N.); maria.mihailescu@upt.ro (M.M.); 2 Faculty of Chemistry, Wroclaw University of Science and Technology, Wybrzeze Wyspianskiego 27, 50-370 Wroclaw, Poland; 3 Research Institute for Renewable Energies, Politehnica University of Timişoara, 138 Gavril Musicescu, 300501 Timisoara, Romania

**Keywords:** diluted waste solution, gold recovery, sorption, sorption mechanism

## Abstract

2,2′-thiobisethanol dimethacrylate/ethylene glycol dimethacrylate copolymer (coP-TEDMA/EGDMA) was used as a sorbent for gold recovery from residual solutions resulting from the electroplating industry. Firstly, synthesized material was characterized by X-ray diffraction, scanning electron microscopy, energy dispersive X-ray spectroscopy, and confocal laser scanning microscopy. The sorption process mechanism was evidenced on the basis of kinetic, thermodynamic and equilibrium studies. To highlight this, the influence of solution pH, temperature and gold initial concentration on maximum sorption capacity was studied. The obtained experimental data were modeled using Langmuir, Freundlich and Sips sorption isotherms, and it was observed that the Sips one was better for describing the studied sorption process. Kinetic data were fitted using pseudo-first-order and pseudo-second-order kinetic models. Of these models, the studied process was better described by the pseudo-second-order model. The thermodynamic parameters free Gibbs energy (ΔG0), enthalpy (ΔH0), and entropy (ΔS0) were evaluated on the basis of the van’t Hoff equation. On the basis of the thermodynamic study, it was concluded that gold recovery on coP-TEDMA/EGDMA is a spontaneous and endothermic process.

## 1. Introduction

Noble metals present a large number of applications in different fields, including as catalysts for chemical and electrochemical processes, for production of electronic devices, as an anticorrosive layer, and in jewelry production [1,2,3,4,5]. 

Global gold resources are decreasing [6], so in this context, it is important to recover gold from different wastes (solid wastes or liquid waste solutions from electroplating industry). Among all of the well-known recovery methods, in the case of gold recovery, the best results are obtained when using oxidation, reduction, precipitation, membrane filtration, ionic exchange and adsorption [1]. Among all of these recovery methods, sorption is the most efficient one, and also has the lowest operating cost. The key factor in adsorptive processes is the sorbent used; the most well-known among these materials are synthetic and natural resins, which, due to the presence of functional groups containing N, S or P atoms, are able to link directly to the metallic ions. Due to the possibility of forming specific interactions between such polymeric resins and metallic ions, such sorbents present a higher selectivity. The main advantage of such polymeric resins is that they can be easily regenerated and reused.

Higher efficiency of recovery processes by sorption on polymeric resins is dependent on the degree of coordination of the resin, the type and structure of the inserted ligand, and on the degree of polymer swelling. Therefore, the metallic ionic properties (ion charging, ionic radius, hydration degree) are important factors in ease of recovery. The recovery process of metallic ions is influenced by various parameters including pH, contact time, temperature and metallic ion concentration [7].

This study aims to establish the gold sorption mechanism on the newly synthesized material: 2,2′-thiobisethanol dimethacrylate/ethylene glycol dimethacrylate (coP-TEDMA/EGDMA) from industrial solutions. This polymer was used due to the presence of divalent sulfur in its structure, so it has the ability to interact with soft acids, such as gold(III) ions, through donor–acceptor interactions.

## 2. Materials and Methods

### 2.1. Preparation of Polymeric Sorbent

The synthesis of 2,2′-thiobisethanol dimethacrylate and ethylene glycol dimethacrylate copolymer (coP-TEDM/EGDMA) was carried out according to the previously published procedures [8]. Briefly, sorbent was synthesized through suspension polymerization using a radical mechanism. The continuous water phase comprised 5% *w*/*w* calcium chloride and 1% *w*/*w* poly(vinyl alcohol) (PVA) (calculated for organic phase). The dispersed organic phase contained monomers, initiator (BPO (0.5% *w*/*w* calculated for monomer)) and solvents (cyclohexanone and n-heptane). The TEDM monomer was obtained in the authors’ laboratory. The process of monomer synthesis and its properties are described in detail in [8]. The second monomer EGDMA was a commercial one (Sigma-Aldrich, Poznan, Poland). The weight ratio of TEDM:EGDMA was 4:1. To create a porous structure in the copolymer, cyclohexanone and n-heptane were used in the weight ratio 1:1 as porogen agents. The ratio of solvents to monomers in the polymerization mixture was 1:1 *w*/*w*. The polymerization took 9 h, and the processing temperature was 60 °C in the first hour, 70 °C in the second hour, 85 °C in the third and fourth hour, and 92 °C in the final five hours. The obtained sorbent was continuously extracted with toluene in a Soxhlet apparatus (ChemLand, Stargard, Poland) to purify it.

### 2.2. Characterization of Polymeric Sorbent

The chemical composition of the investigated sorbent was specified on the basis of elemental analysis. The elemental analyzer (CHNS Model EA1110, CE Instruments, Wigan, UK) was used to determine the sulfur, carbon, hydrogen and oxygen contents in TEDM/EGDMA copolymer. The water regain, W_H_2___O_ (g/g), of the material was determined using the centrifugation method and was calculated using the following equation:

W_H_2___O_ = (*m*_w_ − *m*_d_)/*m*_d_ (1)

where *m_w_* (g) is the weight of the wet sorbent after centrifugation in a small column with a fritted-glass bottom, and *m_d_* (g) is the weight of sorbent after drying at 100 °C overnight. 

The porous structure of the synthesized material was examined by nitrogen adsorption at the liquid nitrogen temperature. The Micromeritics ASAP 2020 analyzer (Micromeritics Instrument Corp., Atlanta, GA, USA) was used. Resultant data were subjected to Brunauer–Emmett–Teller (BET) treatment. 

The produced polymeric sorbent was also characterized using X-ray diffraction (Rigaku Ultima IV diffractometer, Rigaku Corporation, Tokyo, Japan), energy dispersive X-ray spectroscopy (FEI Quanta FEC 250 scanning electron microscope, Hillsboro, OR, USA), and confocal laser scanning microscopy (Olympus OLS 4000 confocal laser 3D microscope, Olympus Corporation, Tokyo, Japan). 

### 2.3. Sorption Studies

The gold-containing solutions used during the experimental determinations were obtained from gold coating processes used in the electronics industry. Residual solutions resulting from the electronics industry, from the industrial process whereby the copper coatings on printed circuit boards are gold plated, were used in this study. They contained K[Au(CN)_2_], as well as cyanide complexes of Au (III), KCN, Ni (II), and Cu (II) [1]. In such residual solutions, gold exists in the form of the cyanide complex, and it is necessary to destroy the cyanide matrix in order to release gold ions for the purpose of their recovery. The cyanide matrix was destroyed when the exhausted gold solution containing 2 g Au (III)/L was treated with an HCl (37 wt.%) and HNO_3_ (63 wt.%) solution [1,2,3].

Solution pH exerts a significant influence on the adsorbent material affinity with respect to the sorption of a specific ion. Solution pH influences the form of the metallic ions in solution and the form of the adsorbent functional groups. Other factors that also have a significant influence on the gold adsorption process include contact time between the adsorbent material and the gold-containing solution and the temperature at which the process takes place.

In the present paper, we studied the influence of pH on the gold sorption process with respect to the obtained sorbent material. This experiment was carried out by varying the solution pH between 1 and 11, for an initial concentration of gold of 10 mg/L, using 0.1 g of adsorbent material, with 2 hours contact time, at a temperature of 298 K.

To establish the effect of contact time and temperature of the sorption process on the maximum sorption capacity of the produced copolymer, exactly 0.1 g of the sorbent material was kept in contact with 25 mL Au(III) solution with an initial concentration of 10 mg/L. Each sample was mixed at 200 rpm for different lengths of time (30, 45, 60, 90, 120 and 240 min) in a thermostatic bath at three different temperatures (298, 308, and 318 K).

The effect of Au(III) initial concentration on copolymer maximum sorption capacity was established by conducting sorption experiments using Au(III) solutions with different concentrations (5, 10, 15, 20, 25, 50, 75, 100, 125, 150, and 175 mg/L). All adsorptive processes were conducted using the previously established values of pH, time, and temperature. In all experimental studies, the residual gold concentrations were determined on the basis of Atomic Absorption Spectroscopy (using a spectrometer Varian SpectrAA 280FS, Varian, Palo Alto, CA, USA).

Sorption capacity was calculated using the relation:

(2) $$q=\frac{\left(C_{0}-C_{f}\right)V}{m}$$

where *q*—sorption capacity (mg/g), *C_0_*—Au(III) initial concentration (mg/L), *C_f_*—Au(III) residual concentration (mg/L), *V*—solution volume (L), *m*—sorbent mass (g).

Equilibrium parameters, generally known as sorption isotherms, were established to better understand the mechanism of the gold sorption process. The sorption process of gold ions onto the produced sorbent material at a constant temperature was modeled using the classical isotherms: Langmuir, Freundlich and Sips. 

The Langmuir sorption isotherm was developed to describe the sorptive processes that take place in the homogenous media, better describing monolayer sorptive processes. Under this assumption, the active sorptive centers are identical, and are uniformly distributed onto the sorptive material surface. In this process, the capacity of one molecule to be sorbed on some active center is not dependent on the occupancy of active neighboring centers. The nonlinear form of the Langmuir isotherm is as follows [4]:

(3) $$q_{e}=\frac{q_{L}K_{L}C_{e}}{1+K_{L}C_{e}}$$

where *q_e_*—equilibrium sorption capacity (mg/g), *C_e_*—metallic ion equilibrium concentration (mg/L), *q*_l_—Langmuir maximum sorption capacity (mg/g), *K_L_*—Langmuir constant.

The main characteristic of the Langmuir isotherm is represented by the dimensionless constant RL, which represents the separation factor or the equilibrium parameter, which can be evaluated on the basis of the following relation:

(4) $$R_{L}=\frac{1}{1+K_{L}C_{0}}$$

where *R_L_*—separation factor, *K_L_*—Langmuir constant (L/mg), *C*_0_—Au(III) initial concentration (mg/L).

The nonlinear Freundlich isotherm [5] can be expressed using the following equation:

(5) qe=KFCe1nF

where *q_e_*—equilibrium sorption capacity (mg/g), *C_e_*—metallic ion concentration in solution (mg/g), *K_F_* and *n_F_*—characteristic constants that are associated with sorbent relative sorption capacity and with sorption intensity.

The Freundlich isotherm assumption suggests that the sorbent surface is heterogeneous, presenting unlimited active centers, resulting in multilayer sorption.

Using the Langmuir and Freundlich isotherms as a starting point, a new sorption isotherm—the Sips isotherm—was developed, which at limit acts like a Langmuir (when *n_s_* is equal with 1) or a Freundlich one (when (*K_s_C_e_*)*^n^^s^* <<< 1). When the sorbate concentration is low, the Sips isotherm is reduced to the Freundlich isotherm, while for higher sorbate concentrations, it is reduced to the Langmuir isotherm. The nonlinear equation of the Sips isotherm is as follows [6]:

(6) qe=qsKsCe1ns1+KsCe1ns

where *q_s_*—maximum sorption capacity (mg/g), *K_s_*—constant linked with the sorption capacity of the sorbent, *n_s_*—heterogeneity factor. On the basis of Sips-isotherm-associated parameters, a dimensionless parameter named separation factor can be evaluated:

(7) Rs=11+Ks C01ns

where *R_s_*—separation factor, *K_s_*—constant linked with the sorption capacity of the sorbent, *n_s_*—heterogeneity factor, *C*_0_—metallic ions initial concentration.

*R_s_* values express the essential characteristics of the Sips isotherm: when the separation factor has values higher then 1, the sorption isotherm has a concave shape, meaning that the sorption is not favorable. When *R_s_* = 1, the sorption isotherm has a linear form, when *R_s_* has values between 0 and 1, the isotherm has a convex form, and the metallic ion sorption is favorable; when *R_s_* = 0, the metallic ion sorption is irreversible.

Kinetic studies were performed by modeling the obtained experimental data using two different kinetic models: pseudo-first-order (Lagergren kinetic model) and pseudo-second-order (Ho and McKay kinetic model) kinetic models.

The mathematical equation associated with the pseudo-first-order model is as follows [7]:

(8) $$\ln\left(q_{e}-q_{t}\right)=\ln q_{e}-k_{1}\;t$$

where *q_e_*—equilibrium sorption capacity (mg/g), *q_t_*—sorption capacity obtained at a specific time *t* (mg/g), *K*_1_—pseudo-first-order speed constant (1/min), *t*—contact time (min).

The mathematical equation that was used to describe the pseudo-second-order model is as follows [9,10]:

(9) $$\frac{t}{q_{t}}=\frac{1}{k_{2}q_{e}^{2}}+\frac{t}{q_{e}}$$

where *q_e_*—equilibrium sorption capacity (mg/g), *q_t_*—sorption capacity obtained at a specific time t (mg/g), *K*_2_—pseudo-second-order speed constant (1/min), *t*—contact time (min).

The pseudo-first-order equation was obtained from the linear representation of ln(*q_e_*−*q_t_*) as a function of time. On the basis of this equation, the value of *K*_1_ can be determined, and the value of sorption capacity (*q*_e,calc_) can be calculated. The pseudo-second-order equation was obtained from the linear representation of *t*/*q_t_* versus time. On the basis of this representation, the values of *K*_2_ and *q*_e,calc_ can be evaluated.

With respect to gold sorption on the newly produced sorbent, the value of activation energy was evaluated by means of the Arrhenius equation, using the value of the speed constant obtained from the pseudo-second-order kinetic model. To evaluate whether gold sorption on produced material was a spontaneous process, the value of free Gibbs energy was determined using the Gibbs-Helmholtz equation [11]. Standard values of enthalpy and entropy were determined on the basis of the van’t Hoff equation, as well as the equation associated with the linear representation of ln*K_d_* versus 1/T. The equilibrium constant *K_d_* represents the ratio between equilibrium sorption capacity (*q_e_*) and equilibrium concentration (*C_e_*).

Samples obtained after gold sorption on the synthesized sorbent were analyzed using scanning electron microscopy (SEM), energy dispersive X-ray spectroscopy (EDX) and confocal laser 3D microscopy.

## 3. Results and Discussion

### 3.1. Characterization of Polymeric Sorbent

#### 3.1.1. Physico-Chemical Characteristics 

**Figure 1:**
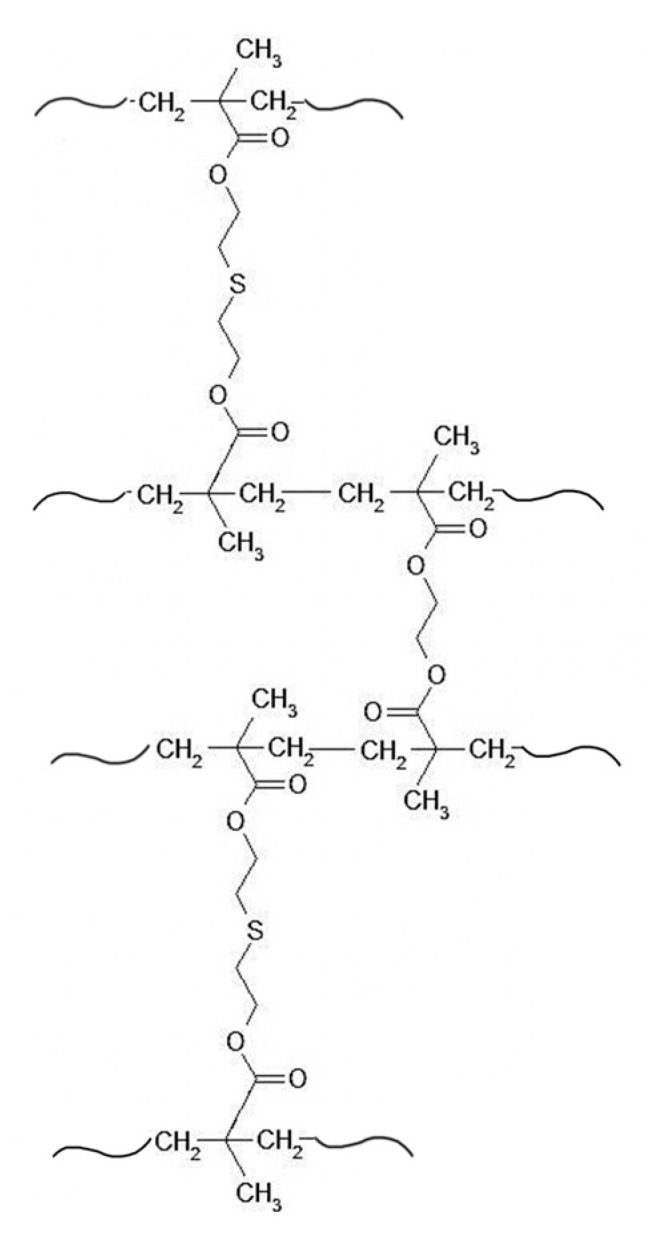
Structure of coP-TEDMA/EGDMA.

A sorptive material possessing coordinating properties and incorporating groups containing donor sulfur atoms in the polymer matrix was proposed for the sorption of gold(III) ions. For this purpose, a copolymer of 2,2′-thiobisethanol dimethacrylate and ethylene glycol dimethacrylate (coP-TEDMA/EGDMA) was synthesized by means of radical suspension polymerization reaction, as described in [8]. During synthesis, the ratio between the monomeric compounds TEDMA:EGDMA was 4:1. The chemical structure of the obtained copolymer is presented in Figure 1. 

The suspension copolymerization of TEDMA and EGDMA yielded polymer grains with an average dimension of between 0.1 and 1 mm. The efficiency of the copolymerization was 80%. The studied polymeric adsorbent presents a moderate surface area of 58 m^2^/g. It has a predominantly mesoporous structure, with an average pore size of 15.1 nm and a total pore volume of 0.22 cm^3^/g. The selected polymer material has good water regain (2.32 g/g), which has a direct impact on sorption efficiency. The donor–acceptor interactions between the sulfur atoms of the sorbent and the gold ions ensure selective sorption. The sulfur content in the tested sorbent, determined on the basis of elemental analysis, is 8.0 wt.%. The oxygen, carbon and hydrogen contents are 27.5 wt.%, 57.6 wt.% and 6.9 wt.%, respectively. The experimental data for elemental analysis are comparable with the atom content obtained theoretically.

#### 3.1.2. Scanning Electron Microscopy, SEM, and Energy Dispersive X-ray Diffraction, EDX

**Figure 2:**
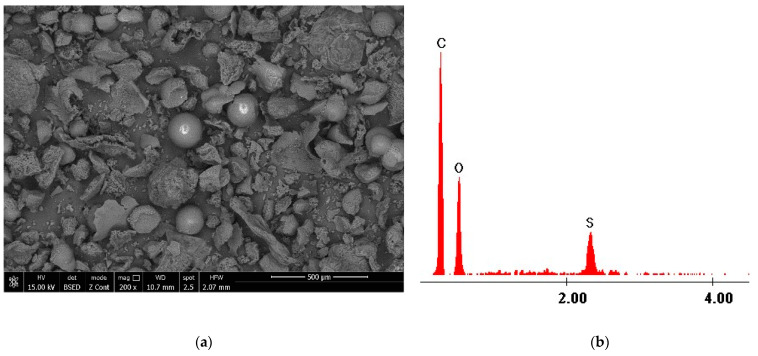
Scanning electron microscopy, SEM, and energy dispersive X-ray, EDX, for coP-TEDMA/EGDMA. (**a**) SEM migrography; (**b**) EDX spectra.

The structure of the coP-TEDMA/EGDM sorbent was investigated by recording scanning electron micrographs, as depicted in Figure 2a. Figure 2b presents the EDX spectra for the synthesized sorbent.

On the basis of an analysis of the micrograph presented in Figure 2a, it is possible to observe the porous structure of the used sorbent, and it can be seen that the polymer grains present a quasi-spherical structure that is relatively uniformly distributed throughout the polymeric mass. In the EDX spectra presented in Figure 2b, the presence of S atoms can be observed alongside that of C and O. The presence of sulfur atoms in the polymer structure has a beneficial effect on the adsorptive process of gold(III) ions. The proposed sorbent has been demonstrated to work well in sorption studies carried out using model solutions of Au(III) ions [12]. The reusability of the sorbent was examined with respect to its sorption performance in consecutive gold(III) ions sorption/desorption cycles. A mixture of 0.8 M thiourea and 3 M HCl was used as eluent. The results of these experiments were presented in an earlier publication [12], and on the basis of the results obtained after five sorption/desorption cycles, it can be concluded that the removal of gold(III) ions is still effective. The applied eluent did not change the properties of the sorbent, and coP-TEDM/EGDMA demonstrated an ability to achieve 100% regeneration for each cycle performed.

#### 3.1.3. X-ray Diffraction, XRD

**Figure 3:**
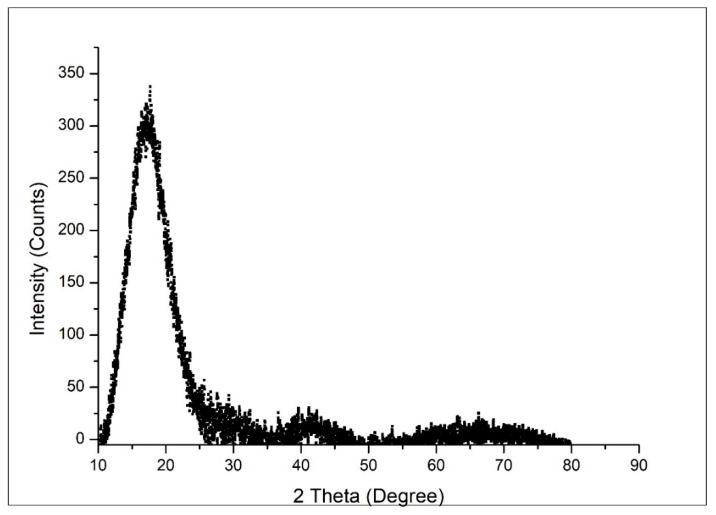
XRD spectrum.

The structure of the produced copolymer was investigated by recording the XRD spectra, as presented in Figure 3.

On the basis of an analysis of the XRD spectra depicted in Figure 3, it can be observed that the produced sorbent material has an amorphous structure; such structures are an advantage with respect to usage as a sorbent material for gold recovery.

#### 3.1.4. Confocal Laser Scanning Microscopy, CLSM

**Figure 4:**
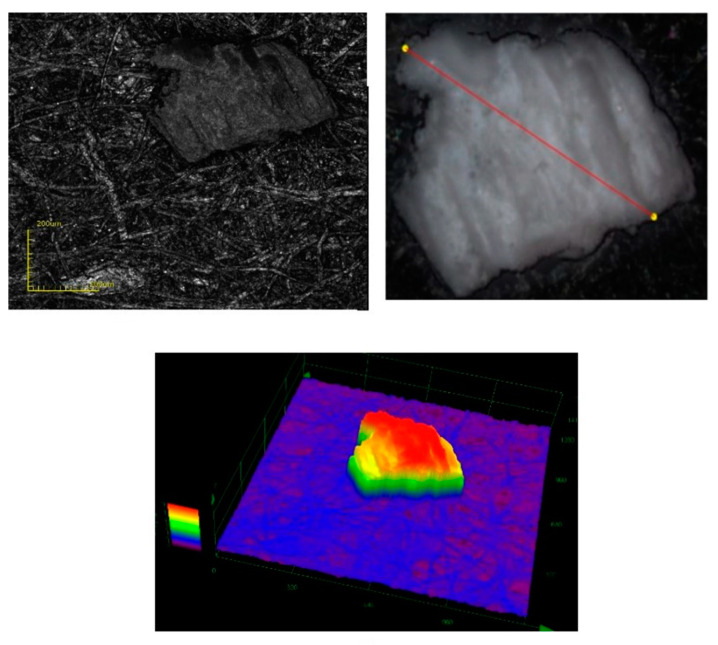
Confocal laser scanning microscopy, CLSM.

Another technique used during the sorptive material characterization process was the confocal laser 3D microscopy. This technique allows the visualization of polymeric grains at a depth of 100 μm, making it possible to obtain, in this way, information regarding the product structure. The recorded images are presented in Figure 4. 

The data presented in Figure 4 demonstrate the amorphous structure of the investigated copolymer grains, as well as making it possible to determine the dimensions of the sorbent particles, which have an average size of 45 μm.

### 3.2. Sorption Studies

#### Effect of pH

**Figure 5:**
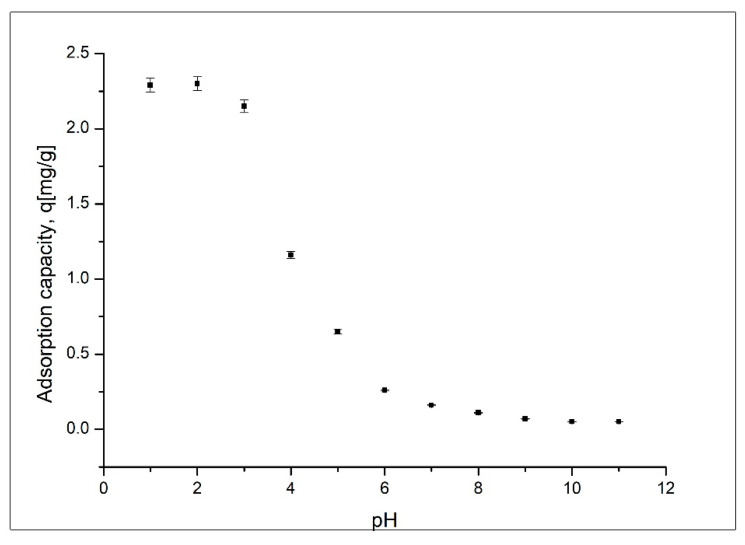
pH effect.

A huge number of experimental studies have demonstrated that one important parameter for adsorptive processes is the solution pH, which can limit the sorption of metallic ions [13]. As a result of this fact, it is important to determine the influence of pH on the sorption of gold ions onto coP-TEDMA/EGDMA copolymeric beads. The obtained experimental results are presented in Figure 5.

pH value is dependent not only on the nature of the sorptive material, but also on the chemical behavior of gold ions with respect to the aqueous solution [13,14,15]. At values of pH lower than 3, due to the presence of HCl, the main species is HAuCl_4_, which dissociates in H^+^ and AuCl_4_^−^ ions [16]. The protons bond with the free electrons of O and S atoms from the polymeric structure, resulting in a surface with a positive charge, on which the AuCl_4_^−^ ions are adsorbed [16]. The number of H^+^ ions adsorbed onto the surface of the adsorbent material decreases simultaneously with the increase of the attractive forces between the positive gold ions and the free active sites from the surface of the material, negatively charged, leading to an increase in the maximum adsorption capacity. When the pH value is low, some competition between H+ and Au (III) ions can be observed [17,18]. The decreased adsorption capacity at higher pH values can be explained by considering that the predominant gold species are less adsorbable [16]. On the basis of the data presented in Figure 5, it can be observed that the maximum value of sorption capacity (approximatively 2.3 mg/g) was obtained when the pH was between 1 and 3. Further increases in pH have negative effects, leading to decreases in the maximum sorption capacity. On the basis of this observation, all further experiments were carried out at a pH lower than 4.

### 3.3. Kinetic Studies

**Figure 6:**
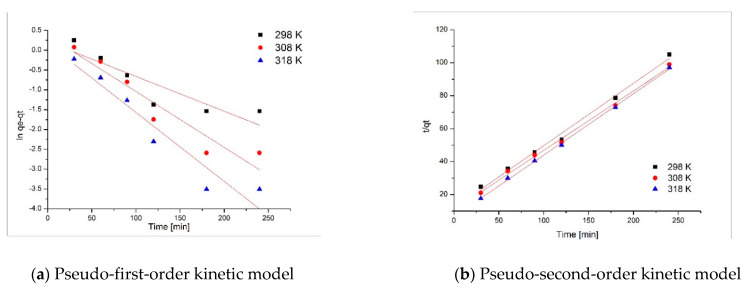
Kinetic studies.

Kinetic studies were carried out by modeling the obtained experimental data using two different models: the pseudo-first-order model (Figure 6a) and the pseudo-second-order model (Figure 6b).

On the basis of the linear dependences presented in Figure 6, the kinetic parameters associated with pseudo-first-order and pseudo-second-order kinetic models were evaluated (parameters presented in Table 1).

On the basis of an analysis of the data presented in Table 1, it can be observed that the regression coefficient R^2^ had a value lower than 1 when the experimental data were modeled using the pseudo-first-order model. In comparison, the regression coefficient value was close to 1 when the experimental data were modeled using the pseudo-second-order model. On the basis of the data presented, it can be observed that the gold (III) adsorption on the coP-TEDMA/EGDMA copolymer is accurately described by the pseudo-second-order kinetic model. This indicates that the speed limit stage is the first sorptive stage, due to the lower time needed for surface saturation [8,9,19]. Can assume that the gold ions sorption is due to the existence of some electrostatic forces between metallic ions and sorbent surface.

**Figure 7:**
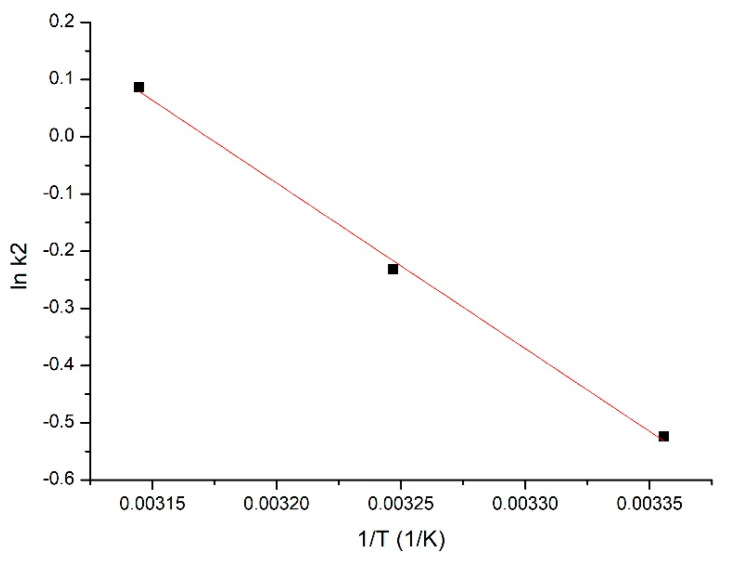
Arrhenius equation plot.

By substituting the obtained speed constant (*k*_2_) into the Arrhenius equation, the value of activation energy for the studied sorptive process can be evaluated. This value was obtained on the basis of the line equation obtained from the representation of dependence lnk_2_ versus 1/*T* (Figure 7). The obtained values for activation energy and correlation coefficient are presented in Table 2.

On the basis of the data presented in Table 2, it can be observed that the activation energy has a value lower than 40 kJ/mol, meaning that the gold (III) sorption onto the produced sorbent is a physical process [20].

### 3.4. Thermodynamic Study 

**Figure 8:**
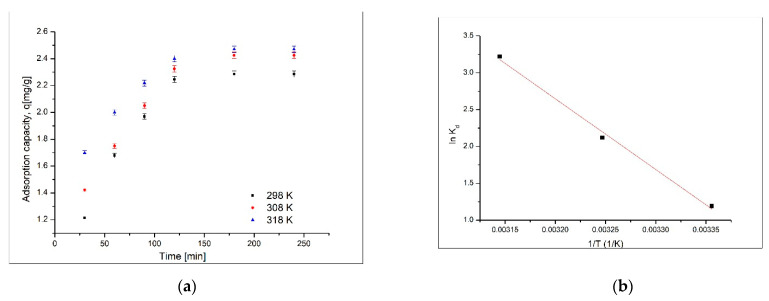
Thermodynamic studies. (**a**) Influence of contact time and temperature; (**b**) Linear dependence ln*k_d_* versus 1/T.

The other two important parameters affecting the sorption of gold (III) ions onto coP-TEDMA/EGDMA copolymer are contact time and temperature. The influence of contact time and temperature is presented in Figure 8a. To establish the energetic modifications associated with the gold sorption, thermodynamic studies were carried out in the temperature interval of 298–318 K. The thermodynamic parameters (free enthalpy (Δ*H*^0^), entropy (Δ*S*^0^) and free Gibbs energy (Δ*G*^0^) variations) indicated the possible nature of the gold adsorption process. On the basis of the linear representation of ln*K*_d_ versus 1/*T* (Figure 8b), the values of entropy and enthalpy variations were evaluated. Starting from these values, by using the van’t Hoff equation, the value of free Gibbs energy variation was calculated.

On the basis of the data presented in Figure 8, it can be observed that the increase of contact time leads to an increase in the maximum sorption capacity until a plateau is reached (this plateau corresponds to the maximum sorption capacity). Simultaneously, it can be observed that the temperature increase exerts a beneficial effect, leading to an increase in the maximum sorption capacity of the material.

The calculated thermodynamic parameters are presented in Table 3.

The negative values of free Gibbs energy variation suggest that the sorption of gold (III) ions is a spontaneous process. Additionally, the decreasing value of free Gibbs energy with increasing of temperature means that gold (III) sorption is favored by increasing temperature. Positive values of the standard enthalpy confirm that the gold sorption process is an endothermic one, which is in concordance with the slow increase of equilibrium sorption capacity and the pseudo-second-order speed constant (*k*_2_). The variation of standard entropy is positive, suggesting that the sorption leads to some disorders at the solid/liquid interface. The fact that the values are low indicates that the changes are major [21].

### 3.5. Effect of Initial Concentration Equilibrium Studies

**Figure 9:**
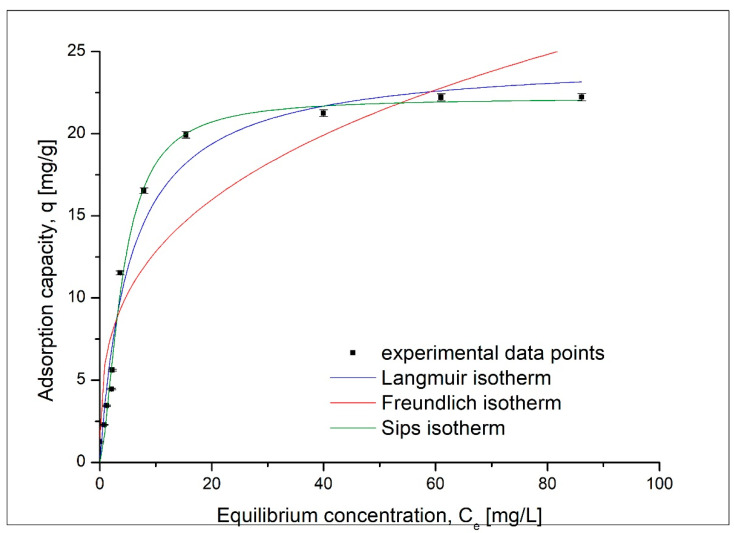
Effect of the initial concentration of Au(III) on sorption capacity.

Another parameter that influences the sorption of gold (III) ions onto the coP-TEDMA/EGDMA copolymer is the initial concentration of gold ions in the solution. The influence of the initial concentration of metal ions is presented in Figure 9.

Based on data presented in Figure 9, it was observed that the increase of the initial concentration of gold has a beneficial effect, leading to an increase in the maximum sorption capacity until saturation of the sorptive material is reached. After that, any further increase in the initial concentration has no effect on the maximum sorption capacity. The maximum sorption capacity of 22.2 mg Au(III)/g of sorbent was obtained with an initial concentration of 150 mg Au(III)/L of solution. In this case, the final concentration was 61 mg Au(III)/L, resulting in a partition coefficient [22,23] of 71.61 mg/g/mM.

**Figure 10:**
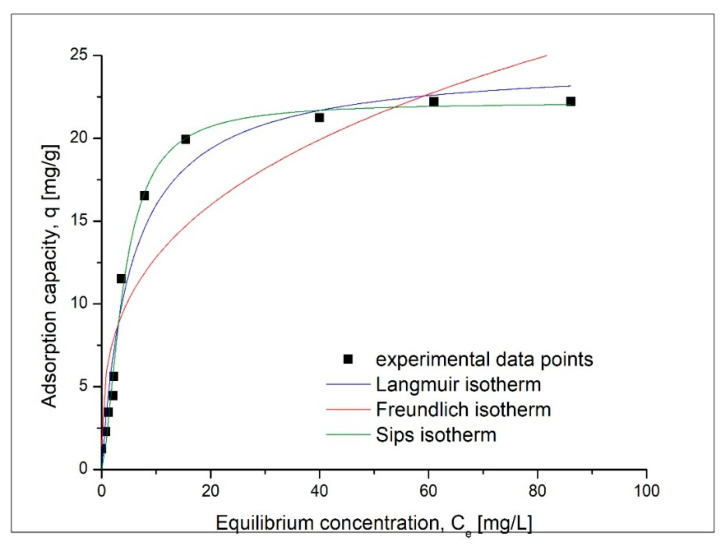
Sorption isotherms models.

To better describe the sorption process of gold(III) ions, the obtained experimental data were fitted using three different sorption isotherms: the Langmuir, Freundlich and Sips isotherms. The sorption isotherms obtained for the sorption of gold(III) ions are presented in Figure 10. On the basis of fitting the experimental data, it can be seen that the Sips isotherm may be applicable for the sorption of gold(III) ions on the analyzed sorbent. The specific parameters were evaluated on the basis of the obtained model sorption isotherms, and these are presented in Table 4.

The highest correlation coefficient (close to 1) obtained for the Sips isotherm could suggest that the Sips model is the most suitable for describing the equilibrium of the sorption of the investigated metal ions [24].

Table 5 presents the maximum sorption capacities obtained for a variety of other materials used as sorbents for gold recovery from aqueous solutions. Compared with other materials, the newly synthesized sorbent had a higher sorption capacity, and therefore it is more efficient for gold recovery.

**Figure 11:**
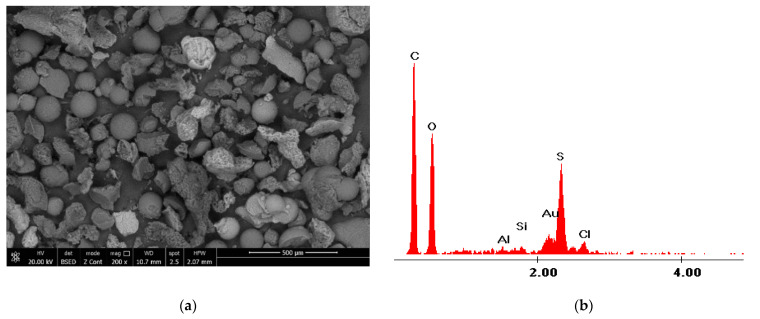
Scanning electron microscopy, SEM and energy dispersive X-ray, EDX, for exhausted coP-TEDMA/EGDMA after gold sorption. (**a**) SEM migrography; (**b**) EDX spectra.

To prove that the gold was sorbed onto the synthesized material, the SEM micrographs and EDX spectra were recorded, as presented in Figure 11.

The obtained SEM image confirms that the gold particles are uniformly distributed on the sorbent surface. The EDX spectra confirm the presence of gold particles on the surface of the used adsorbent material. Other elements present in the spectrum are specific to the support on which the sample is placed for analysis.

## 4. Conclusions

The obtained experimental results demonstrated that the newly synthesized sorbent material with active groups containing sulfur atoms presents higher efficiency with respect to the sorption of gold ions from diluted industrial residual solutions. The maximum sorption capacity was determined to be around 22 mg gold ions/g of sorbent, which was obtained for an initial gold concentration of 150 mg per liter, and a contact time of 120 min.

In the first stage, the cyanuric matrix was broken by treating the industrial residual solution with a mixture of HCl and HNO_3_, resulting in $AuCl_{4}^{-}$. The experimental data proved that the sorption process exhibited its maximum efficiency when the solution pH was between 1 and 3.

The sorption mechanism was evaluated on the basis of kinetic, thermodynamic and equilibrium studies. The sorption of gold ions on the produced sorbent corresponds to the pseudo-second-order kinetic, and was better fitted by the Sips sorption isotherm. The sorption process is favorable, as it takes place as a result of the physical interactions between metallic ions and the active centers of the sorbent material.

To recover metallic gold that can be used directly in different industrial fields (e.g., electronics, medicine, jewelry, and the production of materials with anticorrosive properties), the exhausted sorbent material can be thermally treated by heating it to 873.15 K. 

## Figures and Tables

**Table 1:** Kinetic parameters for the sorption of gold onto the investigated sorbent.

**Pseudo-First Order**				
Temperature (K)	*q*_e,exp_ (mg·g^−1^)	*k*_1_ (min^−1^)	*q*_e,calc_ (mg·g^−1^)	R^2^
298	2.28	0.0088	1.24	0.8121
308	2.42	0.0141	1.45	0.9107
318	2.47	0.0174	1.18	0.924
**Pseudo-Second Order**				
Temperature (K)	*q*_e,exp_ (mg·g^−1^)	*k*_2_ (g mg^−1^·min^−1^)	*q*_e,calc_ (mg·g^−1^)	R^2^
298	2.28	0.591	2.63	0.9942
308	2.42	0.793	2.76	0.9963
318	2.47	1.090	2.67	0.9989

**Table 2:** Activation energy and correlation coefficient values.

Material	Activated Energy, E_a_ kJ/mol	R^2^
(coP-TEDMA/EGDMA)	24.1	0.9982

**Table 3:** Thermodynamic parameters for the sorption of gold onto the investigated sorbent.

Δ*H*º (kJ/mol)	Δ*S*º (J/mol·K)	Δ*G*º (kJ/mol)			R^2^
79.7	2.72	298 K	308 K	318 K	0.9955
		−2.8	−5.6	−8.4	

**Table 4:** Parameters of the isotherm model for the sorption of Au(III) onto the investigated sorbent.

**Langmuir Isotherm**			
*q*_m,exp_ (mg/g)	*K*_L_ (L/mg)	*q*_L_ (mg/g)	*R* ^2^
22.2	0.185	24.6	0.9667
**Freundlich Isotherm**			
*K*_F_ (mg/g)	1/*n*_F_		*R* ^2^
6.16	0.318		0.8465
**Sips Isotherm**			
*K*_S_	*q*_S_ (mg/g)	1/*n*_S_	*R* ^2^
0.63	22.18	0.10	0.9884

**Table 5:** Sorption capacities of some sorbents cited in the literature.

Sorbent	Sorption Capacities, mg Au(III)/g	Reference
3-(8-Quinolinylazo)-4-hydroxybenzoic acid modified nanometer-sized alumina	17.7	[25]
Nanometer TiO2 immobilized on silica gel	3.56	[26]
Amberlite XAD-2000/DDTC	12.3	[27]
2-Mercaptobenzothiazole-bonded silica gel	4.5	[28]
coP-TEDMA/EGDMA	22.2	Present study

## Data Availability

Not applicable.
